# Dynamic Dual-Antenna Time-Slot Allocation Protocol for UAV-Aided Relaying System Under Probabilistic LoS-Channel

**DOI:** 10.3390/s25247443

**Published:** 2025-12-07

**Authors:** Ping Huang, Jie Lin, Tong Liu, Jin Ning, Junsong Luo, Bin Duo

**Affiliations:** The College of Computer Science and Cyber Security, Chengdu University of Technology, Chengdu 610059, China; huangping@stu.cdut.edu.cn (P.H.); liutong1982@cdut.edu.cn (T.L.); ningjin@cdut.edu.cn (J.N.); luojuns@cdut.edu.cn (J.L.); duobin@cdut.edu.cn (B.D.)

**Keywords:** UAV communications, two-way relay, resource scheduling, trajectory optimization, non-convex optimization

## Abstract

Unmanned Aerial Vehicle (UAV)-aided two-way relaying systems have attracted widespread attention due to their ability to improve communication efficiency, reduce deployment costs, and enhance reliability. However, most existing systems employ the Time-Division Multiple Access (TDMA) protocol, which suffers from rigid resource allocation and fails to efficiently manage antenna resources within a time slot for multiple users. Furthermore, the reliance on simple Line-of-Sight (LoS) channel models in many studies is often inaccurate, leading to significant performance degradation. To address these issues, this paper investigates a UAV-assisted two-way relaying system based on the Probabilistic Line-of-Sight (PrLoS) model. We propose a novel two-way transmission protocol, termed the Dynamic Dual-Antenna Time-Slot Allocation Protocol (DDATSAP), to facilitate flexible antenna resource allocation for multiple user pairs. To maximize the minimum average message rate for ground users, we jointly optimize the Resource Scheduling Factor (RSF), transmit power, and UAV trajectory. Since the formulated problem is non-convex and challenging to solve directly, we propose an efficient iterative algorithm based on Successive Convex Approximation (SCA) and Block Coordinate Descent (BCD) techniques. Numerical simulation results demonstrate that the proposed scheme exhibits superior performance compared to benchmark systems.

## 1. Introduction

In recent years, 6G technology has garnered immense interest and undergone rigorous investigation, poised to revolutionize the Unmanned Aerial Vehicle (UAV) sector in the forthcoming era. Its advantages for UAVs are profound and multifaceted, encompassing ultra-high speeds coupled with ultra-low latency, ultra-dense connectivity capabilities, an integrated air–space–ground network architecture, a substantial leap in intelligence levels, and unparalleled enhancements in security and reliability. The advent of 6G technology heralds a new dawn for the UAV field, propelling its development and popularization to unprecedented heights. In pivotal 6G application scenarios, including emergency communications, traffic management, surveillance, agricultural Internet of Things (Agri-IoT) [1], and search and rescue, UAVs, leveraging their exceptional agility, can significantly expand communication footprints, minimize signal transmission losses, elevate communication quality standards, and drastically reduce latency. In the face of natural disasters or combat zones where traditional terrestrial communication infrastructure may be compromised or inaccessible, UAVs can be swiftly deployed to offer vital, temporary communication lifelines, ensuring the continuity of critical transmissions. Furthermore, in recent years, the empowerment of general artificial intelligence large models (LAIMs) has enabled UAVs to realize efficient semantic communication and edge intelligence, thereby significantly enhancing the reliability and efficiency of communication services in dynamic low-altitude environments [2].

As a prominent application within the domain of UAV communication, UAV relaying offers an effective solution for addressing terrestrial infrastructure failures and facilitating the rapid establishment and recovery of communication networks owing to its distinct advantages. Unlike traditional terrestrial base stations (BSs), which rely on fixed infrastructure, UAVs enable rapid, on-demand deployment. This capability renders them particularly suitable for scenarios such as post-disaster communication recovery, temporary hotspot coverage, and coverage gap compensation in remote areas. Furthermore, UAVs can establish Line-of-Sight (LoS) links with ground terminals more readily than terrestrial BSs, as they are less susceptible to blockage by buildings and complex terrain; consequently, they exhibit superior performance regarding link path loss and channel stability. While traditional BSs are responsible for full-stack functions including access, control, and backhaul, relay UAVs predominantly function as flexible, supplementary nodes designed to enhance coverage or forward signals rather than serving as long-term systemic infrastructure.

In the research on UAV relay, existing works are primarily categorized into one-way relaying and two-way relaying based on the transmission mode. Compared to one-way relaying, two-way relaying can theoretically achieve double the spectral efficiency with limited hardware resources through efficient utilization of the communication spectrum. In recent years, research on UAV relay technology has mainly focused on channel modeling, protocol design, and other aspects, as summarized in Table 1.

As can be seen from Table 1, first, the majority of existing research on UAV relay adopts LoS channel modeling, which is inaccurate, especially in areas with severe shadow fading, leading to significant performance degradation [14,15]. Second, most current studies consider the two-way transmission model, indicating that adopting this model can enhance spectrum utilization and improve communication efficiency [16,17,18,19,20,21]. However, only a few works focus on simple two-user two-way transmission protocols, while most multi-user two-way transmission protocols adopt the TDMA protocol, which is often constrained by fixed resource limitations. For instance, insufficiently small time slots can significantly fragment system resources, while excessively large time slots can lead to resource underutilization, thereby affecting system performance. Moreover, in TDMA, a single user often monopolizes a time slot without taking antenna diversity into account, resulting in low resource utilization.

Furthermore, recent works on dual-antenna UAV communication have focused on utilizing hybrid antenna structures to ensure link stability and collaborative efficiency in highly dynamic environments [22]. This not only confirms the hardware feasibility of deploying dual-antenna systems on UAVs but also lays the foundation for further exploring complex resource scheduling strategies based on dual-antenna configurations.

Based on the aforementioned discussion, this paper investigates a UAV-assisted two-way relay system capable of serving multiple user pairs under Probabilistic Line-of-Sight (PrLoS) channel conditions. Designed as an extended communication framework for emergency scenarios, this system facilitates information transmission between trapped survivors and rescue personnel following the destruction of terrestrial base stations. To deliver efficient and high-speed communication services, we propose a novel transmission protocol termed the Dynamic Dual-Antenna Time Slot Allocation Protocol (DDATSAP). This protocol synthesizes the characteristics of existing protocols by incorporating dynamic time slot resource allocation and antenna diversity. By dynamically adjusting the duration and assignment of time slots for individual users, DDATSAP ensures the intelligent distribution of time-slot and antenna resources across varying users and service types, thereby guaranteeing efficient resource utilization.

Given the necessity of maintaining stable and reliable communication connectivity for users in post-disaster areas, this study aims to maximize the minimum average message rate among ground users by jointly optimizing the Resource Scheduling Factor (RSF), transmit power, and flight trajectory of the UAV. However, this formulation results in a non-convex optimization problem. To address this challenge, we propose a novel iterative algorithm based on Successive Convex Approximation (SCA) and Block Coordinate Descent (BCD) techniques. Numerical results demonstrate that the proposed scheme achieves higher communication rates compared to benchmark schemes that rely on static LoS channel modeling or lack optimization for transmit power and RSF. These results indicate that utilizing a more precise channel model alongside the proposed DDATSAP enables a more accurate assessment of environmental states and facilitates superior resource allocation tailored to specific conditions, thereby significantly enhancing system communication performance in complex environments.

The main contributions of this paper are summarized as follows:

- We propose a novel DDATSAP designed for multi-user pair scenarios, which integrates antenna diversity with dynamic resource scheduling to enhance transmission efficiency.
- We formulate a joint optimization problem to maximize the minimum average message rate for ground users by optimizing the UAV’s RSF, transmit power, and flight trajectory under PrLoS channel constraints.
- To solve the resulting non-convex optimization problem, we develop an efficient iterative algorithm utilizing SCA and BCD techniques.
- Extensive numerical results verify that the proposed scheme significantly outperforms benchmark algorithms, demonstrating robustness and superior communication performance in complex post-disaster environments.

## 2. List of Mathematical Symbols and Variables

Given the extensive use of mathematical formulations and symbols throughout this paper, a comprehensive nomenclature table is provided below. Readers are advised to refer to Table 2 for clarity and ease of reference.

## 3. System Model

### 3.1. UAV Movement Model

**Figure 1:**
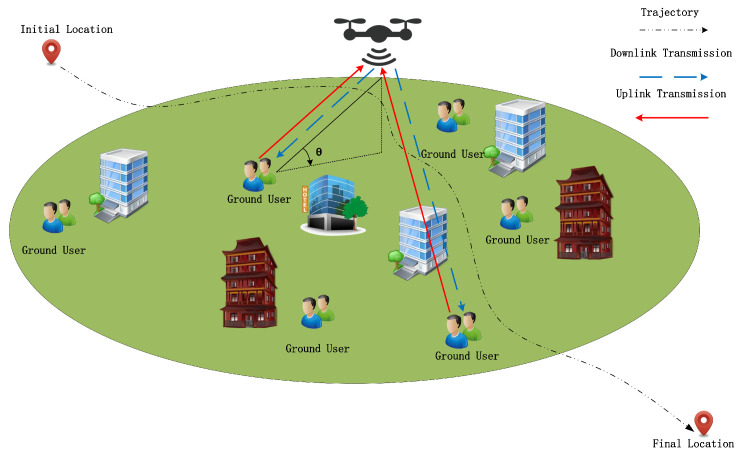
Model of UAV-assisted two-way relaying system.

As illustrated in Figure 1, we consider a UAV-assisted two-way relaying system. Due to temporary communication outages or other unforeseen circumstances, users may find themselves without a communication link prior to the incident where a UAV facilitates the information exchange between multiple pairs of ground nodes (GNs). Assume that the positions of the GNs are fixed, and the horizontal positions of the GNs are denoted as $w_{k}\in R^{2\times 1}$,k∈K≜{1,2…,2∗j}, where *j* is defined as the number of GN pairs.

The time period *T* can be discretized into *I* evenly divided slots, where each duration is $\delta =\frac{T}{I}$. Therefore, the UAV trajectory can be expressed approximately by a length-*I* 3D sequence ${\left\{\left(q[i],h\right)\right\}}_{i=1}^{I}$, where $q\left[i\right]={\left[x\left[i\right],y\left[i\right]\right]}^{T}\in R^{2\times 1}$ and *h* denote the horizontal and vertical coordinates, respectively. We assume the initial and ending positions of the UAV are fixed

(1) $$\;\left(q[1],h\right)=\left(S,h\right)$$

(2) $$\;\left(q[N],h\right)=\left(E,h\right)$$

where (S,h) represents the starting position and (E,h) represents the ending position, and *N* is the last time slot.

To ensure that the proposed system model better aligns with the flight restrictions in real-world environments, this paper assumes that the UAV is subject to flight area limitations during its flight. Specifically, a no-fly zone assumed to be cylindrical is introduced, with its center at point $C={\left[x_{b},y_{b}\right]}^{T}$. To prevent the UAV from violating the no-fly zone (NFZ) regulations during its flight, the distance between the UAV and the center of the no-fly zone must satisfy the following constraint:

(3) $${∥q\left[i\right]-C∥}^{2}\geq r_{b}^{2}$$

where $r_{b}$ is the radius of the no-fly zone.

In addition, the UAV’s maximum horizontal flying speeds, denoted by $\hat{V}$, respectively, in meter/second (m/s), is restricted to the following constraint:

(4) $$∥q\left[i+1\right]-q\left[i\right]∥\leq \hat{\Delta },$$

where $\hat{\Delta }=\delta \hat{V}$ is the maximum horizontal flying distance within each time slot i∈I≜{1,…,I}, respectively. Furthermore, to capture the dominant path loss and shadowing effect on the achieved rate, we model the channel power gain between ground node *k* (GN*k*) and the UAV in each time slot *i* conditioned on the LoS or non-LoS (NLoS) state as $g_{k}^{l}\left[i\right]$ for l∈{L,N}; the channel gains $g_{k}^{L}\left[i\right]$ and $g_{k}^{N}\left[i\right]$ between GN*k* and the UAV at time slot *i* are represented as

(5a) $$\begin{array}{cc} \; & g_{k}^{L}\left[i\right]=\beta_{0}d_{k}^{-\alpha_{L}}\left[i\right],\forall k\in K,\forall i\in I \end{array}$$

(5b) $$\begin{array}{cc} \;\\ \; & g_{k}^{N}\left[i\right]=\mu \beta_{0}d_{k}^{-\alpha_{N}}\left[i\right],\forall k\in K,\forall i\in I \end{array}$$

where μ<1 is the additional signal attenuation factor owing to the NLoS transmission, $\beta_{0}$ denotes the average channel power at a reference distance $d_{0}=1\;m,d_{k}\left[i\right]={\left({∥q\left[i\right]-w_{k}∥}^{2}+h^{2}\right)}^{1/2}$ is the distance between the UAV and GN*k*, and $\alpha_{L}$ and $\alpha_{N}$ represent, respectively, the average path loss exponents for the LoS and NLoS states.

Meanwhile, in this system, it is assumed that the Doppler effect caused by the high-speed flight of the UAV has been effectively compensated and corrected at the receiver of the ground user through advanced signal processing techniques. This ensures that the communication between the UAV and the user remains stable at the receiver end and is not negatively affected by the Doppler effect [23].

### 3.2. DDATSAP Protocol Design

The traditional TDMA protocol generally relies on static time slicing, which often fails to dynamically adjust resource allocation within a time slot to accommodate asymmetric traffic demands or channel variations, leading to resource underutilization. To address this issue, we propose a fine-grained resource management scheme named DDATSAP.

#### 3.2.1. Resource Scheduling Factor (RSF)

We consider a UAV equipped with a dual-antenna full-duplex (DAFD) system serving *j* pairs of ground nodes (GNs). To enable flexible scheduling, we introduce the Resource Scheduling Factor (RSF), denoted as a matrix $A\in R^{4j\times I}$. The matrix A defines the proportion of time within each time slot allocated to different communication links, specifically as follows:

- The first 2j rows represent the proportions of the time slot allocated for downlink transmissions (UAV → Users 1…2j).
- The last 2j rows represent the proportions allocated for uplink transmissions (Users 1…2j to UAV).

Unlike traditional protocols that enforce binary selection (on/off), DDATSAP treats the two antennas as a pooled resource with a total capacity of 2 (dimensionless) per time slot. Consequently, the elements of A represent continuous variables ranging from 0 to 1 rather than discrete time durations.

#### 3.2.2. Dual-Antenna Constraint and Parallelism

While the UAV serves multiple users, the physical limit is determined by its two antennas. DDATSAP allows for the simultaneous execution of operations provided that the total consumed antenna resources do not exceed the hardware limit. Mathematically, this imposes the following constraints on the RSF:

(6a) $$\begin{array}{cc} \; & 0\leq A[r][i]\leq 1,\;\forall i\in \{1,\ldots ,I\},\forall r\in \{1,\ldots ,4j\} \end{array}$$

(6b) $$\begin{array}{cc} \; & \sum_{r=1}^{4j}A\left[r\right]\left[i\right]\leq 2,\;\forall i\in \left\{1,\ldots ,I\right\} \end{array}$$

**Figure 2:**
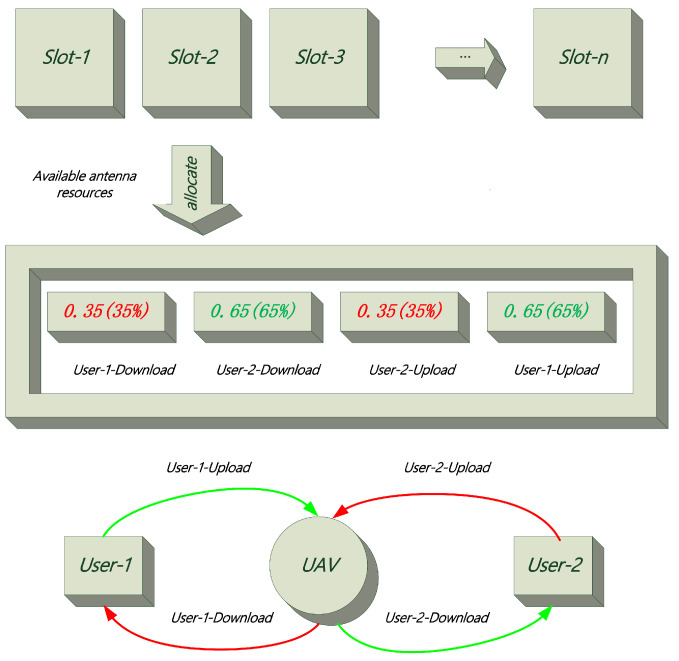
Schematic of DDATSAP: The dual antennas act as a resource pool (Max = 2) dynamically distributed among multiple links.

Constraint (6a) ensures that the allocated time proportion for any single link does not exceed the slot duration δ. Constraint (6b) enforces the physical limitation of the dual-antenna system: the sum of active time proportions for all uplink and downlink links within a slot cannot exceed 2 (i.e., the capacity of two antennas working in full-duplex mode). For example, in Figure 2 if j=1 (two users), a feasible allocation column could be ${\left[0.35,0.65,0.35,0.65\right]}^{T}$, summing to 2. This implies that the dual antennas are fully utilized throughout the time slot to support these four links in a partially overlapping manner, leveraging the full-duplex capability.

#### 3.2.3. Buffering and Information Causality

The UAV functions as a decode-and-forward (DF) relay equipped with an information buffer. This buffer stores uplink messages from GNs before they are forwarded. While the buffer capacity is assumed to be sufficiently large to prevent overflow, the relaying process must strictly adhere to the information causality constraint. This implies that the cumulative data transmitted to a destination node cannot exceed the cumulative data received from the source node up to that time slot. Although the buffer is a logical entity, its operation is mathematically integrated into the optimization problem via the causality constraint derived later in Equation (9). This constraint couples the uplink and downlink RSF variables across time slots, ensuring a valid data flow.

Distinct from rigid TDMA schemes found in [3,7], where resources are orthogonally divided and often underutilized, DDATSAP’s continuous RSF matrix A enables a “soft” allocation. By allowing ∑A[r][i]≤2, the protocol can dynamically balance uplink and downlink traffic loads, significantly improving spectral efficiency in asymmetric traffic scenarios.

### 3.3. Transmission Rate Design

Regarding the communication interface, the system adopts a practical layered architecture that effectively decouples the data plane from the control plane [24,25]. For high-rate data transmission, the system employs an Orthogonal Frequency Division Multiplexing (OFDM)-based interface operating in the sub-6 GHz band. At the physical (PHY) layer, Adaptive Modulation and Coding (AMC) is implemented to mitigate channel fading, thereby validating the use of the Shannon capacity formula for achievable rate analysis; meanwhile, the Medium Access Control (MAC)-layer operations are governed by the proposed DDATSAP to dynamically manage dual-antenna resources.

According to the PrLoS channel model, we have the uplink transmission rate $R_{U_{k}}\left[i\right]$ in kbps from GN*k* to the UAV as

(7) $$R_{U_{k}}\left[i\right]=A\left[u\right]\left[i\right]B\log_{2}\left(1+\frac{p_{U_{k}}\left[i\right]g_{k}^{l}\left[i\right]}{\sigma^{2}\;+\alpha \;p_{D_{\bar{k}}}\left[i\right]}\right)$$

where ∀k∈K,∀u∈{2j+1,…,4j}. Here, α is the residual self-interference (RSI) suppression coefficient at the UAV, modeling the portion of UAV transmit power that leaks into its own receiver. The term $\alpha p_{D_{\bar{k}}}\left[i\right]$ thus represents the RSI at the UAV when it transmits with power $p_{D_{\bar{k}}}\left[i\right]$. We define *k* and $\bar{k}$ as two opposing representations in a pair of users. If k=1, $\bar{k}=2$ and k=2, $\bar{k}=1$ for multiple pairs of users, and so on. And we define *u* and *d* to denote two user numbers in a pair of GNs for RSF. And the mapping rule for *u* and *d* is d=u−2∗j, $\sigma^{2}$ is the power spectral density, and $p_{U_{k}}\left[i\right]$ equals the uplink transmit power of GN*k*.

We assume that each GN can perform RSI cancellation to remove interference induced by its own transmit data. Thus, the downlink transmission rate $R_{D_{k}}\left[i\right]$ at time slot *i* from the UAV to GN*k* is denoted by

(8) $$R_{D_{k}}\left[i\right]=A\left[d\right]\left[i\right]B\log_{2}\left(1+\frac{p_{D_{k}}\left[i\right]g_{k}^{l}\left[i\right]}{\sigma^{2}}\right)$$

where $p_{D_{k}}\left[i\right]$ is the transmitting power from the UAV for GN*k* during the downlink and ∀k∈K,∀d∈{1,…,2*j}.

In data relaying, the information causality implies that at each slot *i*, the relay is able to only transmit the information which has already been collected from the GNs. When the processing delay at the UAV is assumed smaller than the duration of the time slot δ, the information causality constraint can be expressed as

(9) $$\sum_{n=2}^{i}E\left[R_{D_{k}}\left[n\right]\right]\leq \sum_{n=1}^{i-1}E\left[R_{U_{\bar{k}}}\left[n\right]\right],\forall i\in \left\{2,\ldots ,I\right\}$$

In practice, both the GNs and the UAV are typically battery-powered devices with limited energy storage. We consider the average and the peak power constraints both at the GNs and the UAV. Denoting $P_{U,\;\mathrm{avg}\;}$ and $P_{D,\;\mathrm{avg}\;}$ as the average power budgets for the GNs and the UAV, respectively, we have

(10) $$\frac{1}{I-1}\sum_{i=1}^{I-1}p_{U_{k}}\left[i\right]\leq P_{U,\mathrm{avg}},\forall k\in K$$

(11) $$\frac{1}{I-1}\sum_{i=2}^{I}p_{D_{k}}\left[i\right]\leq P_{D,\mathrm{avg}},\forall k\in K$$

Also, when $P_{U,\;\mathrm{peak}\;}$ and $P_{D,\;\mathrm{peak}\;}$ represent the peak power constraints at the GNs and the UAV, respectively, the peak power constraints can be written by

(12) $$0\leq p_{U_{k}}\left[i\right]\leq P_{U,\;\mathrm{peak}\;},\forall k\in K,\forall i\in I$$

(13) $$0\leq p_{D_{k}}\left[i\right]\leq P_{D,\;\mathrm{peak}\;},\forall k\in K,\forall i\in I$$

To focus on characterizing the performance limit of the considered two-way relaying, we assume that the trajectory design is implemented at a central controller in an offline manner (i.e., before the UAV’s mission). We adopt the practical PrLoS channel model for the ground-to-air communication links. Specifically, for GN*k* in time slot *i*, the LoS probability is expressed as

(14) $$P_{k}^{L}\left[i\right]=\frac{1}{1+ae^{\left(-b\left[\theta_{k}\left[i\right]-a\right]\right)}}$$

where a>0 and b>0 are constants specified by the practical environment, and 

(15) $$\theta_{k}\left[i\right]=\frac{180}{\pi }\arctan\left(\frac{h}{∥q\left[i\right]-w_{k}∥}\right)$$

A is the elevation angle from GN*k* to the UAV in the *i*-th slot. The corresponding NLoS probability can then be obtained as

(16) $$P_{k}^{N}\left[i\right]=1-P_{k}^{L}\left[i\right].$$

In a statistical sense, the expected rate under the PLC model from UAV to GN in time slot *i* is given by

(17) $$E\left[R_{D_{k}}\left[i\right]\right]=P_{k}^{L}\left[i\right]R_{D_{k}}^{L}\left[i\right]+P_{k}^{N}\left[i\right]R_{D_{k}}^{N}\left[i\right]$$

where

(18) $$R_{D_{k}}^{L}\left[i\right]=A\left[d\right]\left[i\right]B\log_{2}\left(1+\frac{p_{D_{k}}\left[i\right]\;g_{k}^{L}\left[i\right]}{\sigma^{2}}\right),\;\forall k\in K$$

(19) $$R_{D_{k}}^{N}\left[i\right]=A\left[d\right]\left[i\right]B\log_{2}\left(1+\frac{p_{D_{k}}\left[i\right]\;g_{k}^{N}\left[i\right]}{\sigma^{2}}\right),\;\forall k\in K$$

## 4. Problem Formulation

It is worth noting that we adopt the max-min average rate as the optimization objective rather than maximizing the sum rate or imposing strict instantaneous Quality of Service (QoS) constraints, based on the specific requirements of emergency rescue missions. First, in post-disaster scenarios, fairness is synonymous with survival chances. A sum-rate maximization approach would typically allocate resources to users with strong channel conditions while starving those in deep fading (e.g., trapped under debris), which is unacceptable for search-and-rescue operations. Maximizing the minimum rate ensures that the system provides a robust communication lifeline even to the worst-case user. Second, due to the high mobility of the UAV and the probabilistic nature of the air-to-ground channel (PrLoS), the instantaneous channel capacity fluctuates severely. Imposing strict instantaneous QoS constraints (e.g., a minimum rate per time slot) often leads to infeasibility in optimization when the UAV is temporarily blocked. Instead, the average rate metric guarantees that over the duration of the mission, every user receives a sufficient volume of data to transmit essential information, effectively acting as a long-term QoS guarantee for mission-critical reliability.

In this paper, the transmit power at the UAV $P≜\{p_{o}\left[i\right],o\in M,\forall i\}$, where $M≜\left\{U_{k},U_{\bar{k}},D_{k},D_{\bar{k}}\right\}$, the horizontal trajectory of the UAV Q≜{q[i],∀i}, and the scheduling $S≜\left\{A[u][i],A[d][i],\forall i\right\}$ are jointly optimized to solve the minimum average rate maximization problem between the GNs. Denoting O≜{P,Q,S} as all optimization variables, we define $\eta =\min_{k\in K}\frac{1}{I}\sum_{i=2}^{I}E\left[R_{D_{k}}\left[i\right]\right]$. The optimization problem is formulated as follows:

(P1) Objective:

(20) $$\max_{O}\;\eta$$

subject to

(21) $$\frac{1}{I}\sum_{i=2}^{I}\left(P_{k}^{L}\left[i\right]R_{D_{k}}^{L}\left[i\right]+P_{k}^{N}\left[i\right]R_{D_{k}}^{N}\left[i\right]\right)\geq \eta ,\;\forall k.$$

(22) $$\sum_{n=2}^{i}E\;\left[R_{D_{k}}\left[n\right]\right]\leq \sum_{n=1}^{i-1}E\;\left[R_{U_{\bar{k}}}\left[n\right]\right],\;\;i=2,\ldots ,I.$$

and (1), (2), (3), (4), (6), (10), (11), (12), (13) hold.

Since (P1) involves highly non-convex constraints, obtaining an optimal solution is computationally intractable via standard methods. Consequently, we develop a new optimization algorithm to solve it.

## 5. Proposed Algorithm

In this section, we propose an efficient algorithm for solving (P1) based on the BCD technique, which iteratively solves the approximated problem of (P1). Specifically, we first divide the entire optimization problem into three blocks, namely, scheduling variable S, transmission power P, and UAV trajectory Q. Then, we optimize each variable block with the other two blocks being fixed.

### 5.1. Scheduling Optimization

For any given UAV horizontal trajectory Q and transmit power P, the scheduling problem (P1.1) is as follows:

(P1.1) Scheduling subproblem:

(23) $$\max_{S}\;\eta$$

subject to

(24) $$\frac{1}{I}\sum_{i=2}^{I}E\;\left[R_{D_{k}}\left[i\right]\right]\geq \eta ,\;\forall k.$$

(25) $$\sum_{n=2}^{i}E\;\left[R_{D_{k}}\left[n\right]\right]\leq \sum_{n=1}^{i-1}E\;\left[R_{U_{\bar{k}}}\left[n\right]\right],\;\;i=2,\ldots ,I.$$

and (6).

Where (24) and (25) can be expanded as

(26) $$\begin{array}{cc} \sum_{n=2}^{i}E\;\left[R_{D_{k}}\left[n\right]\right]=\sum_{n=2}^{i}( & A\left[d\right]\left[n\right]B\;P_{k}^{L}\left[n\right]\;\log_{2}\;\left(1+\frac{p_{D_{k}}\left[n\right]g_{k}^{L}\left[n\right]}{\sigma^{2}}\right)\\ \; & \;+A\left[d\right]\left[n\right]B\;P_{k}^{N}\left[n\right]\;\log_{2}\;\left(1+\frac{p_{D_{k}}\left[n\right]g_{k}^{N}\left[n\right]}{\sigma^{2}}\right)). \end{array}$$

(27) $$\begin{array}{cc} \sum_{n=1}^{i-1}E\;\left[R_{U_{\bar{k}}}\left[n\right]\right]=\sum_{n=1}^{i-1}( & A\left[u\right]\left[n\right]B\;P_{\bar{k}}^{L}\left[n\right]\;\log_{2}\;\left(1+\frac{p_{U_{\bar{k}}}\left[n\right]g_{\bar{k}}^{L}\left[n\right]}{\sigma^{2}+\alpha \;p_{D_{k}}\left[n\right]}\right)\\ \; & \;+A\left[u\right]\left[n\right]B\;P_{\bar{k}}^{N}\left[n\right]\;\log_{2}\;\left(1+\frac{p_{U_{\bar{k}}}\left[n\right]g_{\bar{k}}^{N}\left[n\right]}{\sigma^{2}+\alpha \;p_{D_{k}}\left[n\right]}\right)). \end{array}$$

It is straightforward that problem (P1.1) is a convex optimization problem with respect to S, which can be efficiently solved by standard convex optimization solvers such as CVX.

### 5.2. Transmit Power Optimization

For any given UAV horizontal trajectory Q and scheduling S, the transmit-power subproblem is written as

(P1.2) Power subproblem:

(28) $$\max_{P}\;\eta$$

subject to:

(29) $$\frac{1}{I}\sum_{i=2}^{I}E\;\left[R_{D_{k}}\left[i\right]\right]\geq \eta ,\;\forall k.$$

(30) $$\sum_{n=2}^{i}E\;\left[R_{D_{k}}\left[n\right]\right]\leq \sum_{n=1}^{i-1}E\;\left[R_{U_{\bar{k}}}\left[n\right]\right],\;\;i=2,\ldots ,I.$$

and (10), (11), (12), (13).

To incorporate the RSI at the UAV, consider the uplink LoS and NLoS rates in time slot *i*:

(31) $$R_{U_{k}}^{L}\left[i\right]=A\left[u\right]\left[i\right]B\;\log_{2}\;\left(1+\frac{p_{U_{k}}\left[i\right]\;g_{k}^{L}\left[i\right]}{\sigma^{2}+\alpha \;p_{D_{\bar{k}}}\left[i\right]}\right)$$

(32) $$R_{U_{k}}^{N}\left[i\right]=A\left[u\right]\left[i\right]B\;\log_{2}\;\left(1+\frac{p_{U_{k}}\left[i\right]\;g_{k}^{N}\left[i\right]}{\sigma^{2}+\alpha \;p_{D_{\bar{k}}}\left[i\right]}\right)$$

with corresponding expectation

(33) $$E\;\left[R_{U_{k}}\left[i\right]\right]=P_{k}^{L}\left[i\right]R_{U_{k}}^{L}\left[i\right]+P_{k}^{N}\left[i\right]R_{U_{k}}^{N}\left[i\right].$$

Because the denominator $\sigma^{2}+\alpha p_{D_{\bar{k}}}\left[i\right]$ couples uplink power $p_{U_{k}}$ with downlink power $p_{D_{\bar{k}}}$, constraint (30) is non-convex. We introduce slack variables $w_{k}\left[i\right]$ satisfying

(34) $$w_{k}\left[i\right]\leq E\;\left[R_{D_{k}}\left[i\right]\right],\;\forall i,$$

and rewrite the average-rate constraint as

(35) $$\frac{1}{I}\sum_{i=2}^{I}w_{k}\left[i\right]\geq \eta ,\;\forall k.$$

This yields an equivalent reformulation:

(P1.3) Power subproblem reformulation:

(36) $$\max_{P,w}\;\eta$$

subject to

(37) $$\frac{1}{I}\sum_{i=2}^{I}w_{k}\left[i\right]\geq \eta ,\;\forall k.$$

(38) $$\sum_{n=2}^{i}w_{k}\left[n\right]\leq \sum_{n=1}^{i-1}E\;\left[R_{U_{\bar{k}}}\left[n\right]\right],\;\;i=2,\ldots ,I.$$

(39) $$w_{k}\left[i\right]\leq E\;\left[R_{D_{k}}\left[i\right]\right],\;\forall i.$$

and (10), (11), (12), and (13).

To convexify the non-convex dependence of (38), we define a generic uplink component

$$\phi \left(p_{U},p_{D}\right)=AB\log_{2}\;\left(1+\frac{p_{U}g}{\sigma^{2}+\alpha p_{D}}\right).$$

and at iteration *r*, we denote $\left(p_{U}^{r},p_{D}^{r}\right)$ and $t^{r}=\sigma^{2}+\alpha p_{D}^{r}$. The gradients are

(40) $$\begin{array}{cc} {\left.\frac{𝜕\phi }{𝜕p_{U}}\right|}_{r} & =\frac{AB}{\ln2}\frac{g}{t^{r}+p_{U}^{r}g}, \end{array}$$

(41) $$\begin{array}{cc} {\left.\frac{𝜕\phi }{𝜕p_{D}}\right|}_{r} & =-\frac{AB}{\ln2}\frac{\alpha p_{U}^{r}g}{\left(t^{r}+p_{U}^{r}g\right)t^{r}}. \end{array}$$

A global affine upper bound is obtained through first-order Taylor approximation:

(42) $$\phi \left(p_{U},p_{D}\right)\leq \phi^{r}+{\left.\frac{𝜕\phi }{𝜕p_{U}}\right|}_{r}\;\left(p_{U}-p_{U}^{r}\right)+{\left.\frac{𝜕\phi }{𝜕p_{D}}\right|}_{r}\;\left(p_{D}-p_{D}^{r}\right).$$

and weighted LoS/NLoS expansions produce

$${\hat{E}}^{\;lb,r}\;\left[R_{U_{\bar{k}}}\left[n\right]\right],$$

an affine surrogate that replaces $E[R_{U_{\bar{k}}}\left[i\right]]$ in (38).

Thus, for each *i*,

(43) $$\sum_{n=2}^{i}w_{k}\left[n\right]\leq \sum_{n=1}^{i-1}{\hat{E}}^{\;lb,r}\;\left[R_{U_{\bar{k}}}\left[n\right]\right].$$

Replacing the non-convex terms with their affine lower surrogates, the subproblem solved at iteration *r* is as follows:

(P1.4) Power subproblem iterative subproblem:

(44) $$\max_{P,w,\eta }\;\eta$$

subject to

(45) $$\frac{1}{I}\sum_{i=2}^{I}w_{k}\left[i\right]\geq \eta ,\;\forall k.$$

(46) $$\sum_{n=2}^{i}w_{k}\left[n\right]\leq \sum_{n=1}^{i-1}{\hat{E}}^{\;lb,r}\;\left[R_{U_{\bar{k}}}\left[n\right]\right],\;\;i=2,\ldots ,I.$$

(47) $$w_{k}\left[i\right]\leq E\;\left[R_{D_{k}}\left[i\right]\right],\;\forall i.$$

and (10), (11), (12), and (13).

This is a convex optimization problem and can be solved efficiently using CVX. The SCA procedure then updates (P,w) iteratively until convergence.

### 5.3. Horizontal Trajectory Optimization

For any given scheduling S, transmit power P, we have the following:

(P1.5) Trajectory subproblem:

(48) $$\max_{Q}\;\eta$$

subject to

(49) $$\frac{1}{I}\sum_{i=2}^{I}E\;\left[R_{D_{k}}\left[i\right]\right]\geq \eta ,\;\forall k.$$

(50) $$\sum_{n=2}^{i}E\;\left[R_{D_{k}}\left[n\right]\right]\leq \sum_{n=1}^{i-1}E\;\left[R_{U_{\bar{k}}}\left[n\right]\right],\;\;i=2,\ldots ,I.$$

and (1), (2), (3), and (4). We unfold (49) as follows:

(51) $$\begin{array}{cc} E\;\left[R_{D_{k}}\left[i\right]\right] & =P_{k}^{L}\left[i\right]\;A\left[d\right]\left[i\right]B\;\log_{2}\;\left(1+\frac{p_{D_{k}}\left[i\right]\beta_{0}{\left(∥q\left[i\right]-w_{k}{∥}^{2}+h^{2}\right)}^{-\alpha_{L}/2}}{\sigma^{2}}\right)\\ \; & \;+\;P_{k}^{N}\left[i\right]\;A\left[d\right]\left[i\right]B\;\log_{2}\;\left(1+\frac{p_{D_{k}}\left[i\right]\mu \beta_{0}{\left(∥q\left[i\right]-w_{k}{∥}^{2}+h^{2}\right)}^{-\alpha_{N}/2}}{\sigma^{2}}\right). \end{array}$$

We define $\gamma_{1,F_{m}}=\frac{\beta_{0}p_{F_{m}}\left[i\right]}{\sigma^{2}},\forall i\in I,F\in \left\{D,U\right\}$, $m\in \{k,\bar{k}\}$ and introduce relaxation variables $x_{mk}$, ∀m∈{1,2} and $y_{1k,l}$, ∀l∈{L,N} to further simplify the expression. Then $E[R_{D_{k}}\left[i\right]]$ in (49) can be rewritten as

(52) $$\frac{1}{BA[d][i]}E\;\left[R_{D_{k}}\left[i\right]\right]=\frac{1}{x_{1k}\left[i\right]}\log_{2}\;\left(1+\gamma_{1,D_{k}}\;y_{1k,L}^{-1}\left[i\right]\right)+\frac{1}{x_{2k}\left[i\right]}\log_{2}\;\left(1+\gamma_{1,D_{k}}\mu \;y_{1k,N}^{-1}\left[i\right]\right)$$

with the following additional constraints:

(53) $$x_{1k}\left[i\right]=1+a\;e^{-b\;[\theta_{1,k}\left[i\right]-a]}$$

(54) $$x_{2k}\left[i\right]=1+\frac{1}{a}e^{b\;[\theta_{1,k}\left[i\right]-a]}$$

Considering the non-convex constraints (53) and (54), we introduce relaxation variables $z_{1k}\left[i\right]$ and $z_{2k}\left[i\right]$ and apply first-order Taylor expansions at given points $z_{1k}^{r}\left[i\right]$ and $z_{2k}^{r}\left[i\right]$ in iteration *r*:

(55) $$x_{1k}\left[i\right]\leq 1+a\;e^{-b\;z_{1k}^{r}\left[i\right]}-ab\;e^{-b\;z_{1k}^{r}\left[i\right]}\left(z_{1k}\left[i\right]-z_{1k}^{r}\left[i\right]\right)$$

(56) $$x_{2k}\left[i\right]\leq 1+\frac{1}{a}e^{b\;z_{2k}^{r}\left[i\right]}+\frac{b}{a}e^{b\;z_{2k}^{r}\left[i\right]}\left(z_{2k}\left[i\right]-z_{2k}^{r}\left[i\right]\right)$$

with additional constraints:

(57) $$z_{1k}\left[i\right]\geq \frac{180}{\pi }\arctan\left(\frac{h}{∥q\left[i\right]-w_{k}∥}\right)-a$$

(58) $$z_{2k}\left[i\right]\leq A_{k}^{r}\left[i\right]-\left(∥q\left[i\right]-w_{k}∥-∥q^{r}\left[i\right]-w_{k}∥\right)\;B_{k}^{r}\left[i\right]$$

where $A_{k}^{r}\left[i\right]$ and $B_{k}^{r}\left[i\right]$ are constants given by

(59) $$A_{k}^{r}\left[i\right]=\frac{180}{\pi }\arctan\left(\frac{h}{∥q^{r}\left[i\right]-w_{k}∥}\right)-a$$

(60) $$B_{k}^{r}\left[i\right]=\frac{180}{\pi }\cdot \frac{h}{∥q^{r}\left[i\right]-w_{k}{∥}^{2}+h^{2}}\;$$

After the above processing, (52) is still complex. To handle the terms, $\frac{1}{BA[d][i]}E\;\left[R_{D_{k}}\left[i\right]\right]$ can be equivalently expressed as

(61) $$\frac{1}{BA[d][i]}E\;\left[R_{D_{k}}\left[i\right]\right]=\Omega_{5,k}\left[i\right]+\Omega_{6,k}\left[i\right]$$

with additional constraints

(62) $$\left\{\begin{array}{c} \Omega_{5,k}\left[i\right]\geq \frac{1}{x_{1k}\left[i\right]}\left(\Lambda_{5}\left[i\right]+1\right),\;\;\Lambda_{5}\left[i\right]\geq \log_{2}\left(1+\frac{\gamma_{1,D_{k}}}{y_{1k,L}\left[i\right]}\right)-1,\\ \Omega_{6,k}\left[i\right]\geq \frac{1}{x_{2k}\left[i\right]}\left(\Lambda_{6}\left[i\right]+1\right),\;\;\Lambda_{6}\left[i\right]\geq \log_{2}\left(1+\frac{\gamma_{1,D_{k}}\mu }{y_{1k,N}\left[i\right]}\right)-1 \end{array}\right.$$

where the equality must hold at optimum. To linearize the positive variables, we introduce $v_{5}\left[i\right]$ and $v_{6}\left[i\right]$ and obtain

(63) $$\Lambda_{5}\left[i\right]\geq \log_{2}\left(1+e^{v_{5}\left[i\right]}\right),\;\Lambda_{6}\left[i\right]\geq \log_{2}\left(1+e^{v_{6}\left[i\right]}\right)$$

(64) $$v_{5}\left[i\right]\geq \log\left(\frac{\gamma_{1,D_{k}}\left[i\right]}{y_{1k,L}\left[i\right]}\right),\;v_{6}\left[i\right]\geq \log\left(\frac{\mu \;\gamma_{1,D_{k}}\left[i\right]}{y_{1k,N}\left[i\right]}\right)$$

At iteration *r*, a first-order Taylor expansion yields global affine lower bounds

(65) $$\begin{array}{cc} {\hat{\Lambda }}_{5}^{lb,r}\left[i\right]\; & =\log_{2}\left(1+e^{v_{5}^{r}\left[i\right]}\right)+\frac{e^{v_{5}^{r}\left[i\right]}}{\ln2\;\left(1+e^{v_{5}^{r}\left[i\right]}\right)}\left(v_{5}\left[i\right]-v_{5}^{r}\left[i\right]\right),\\ {\hat{\Lambda }}_{6}^{lb,r}\left[i\right]\; & =\log_{2}\left(1+e^{v_{6}^{r}\left[i\right]}\right)+\frac{e^{v_{6}^{r}\left[i\right]}}{\ln2\;\left(1+e^{v_{6}^{r}\left[i\right]}\right)}\left(v_{6}\left[i\right]-v_{6}^{r}\left[i\right]\right) \end{array}$$

(66) $$\Lambda_{5}\left[i\right]\geq {\hat{\Lambda }}_{5}^{lb,r}\left[i\right],\;\Lambda_{6}\left[i\right]\geq {\hat{\Lambda }}_{6}^{lb,r}\left[i\right]$$

and the right-hand side of (50) is convex with respect to the corresponding variable. We define $\gamma_{2,F_{m}}=\frac{\beta_{0}p_{F_{m}}\left[i\right]}{\sigma^{2}+\alpha \;p_{D_{\bar{m}}}\left[i\right]},\forall i\in I,F\in \left\{D,U\right\}$, $m\in \{k,\bar{k}\}$ and introducing relaxation variables $x_{m\bar{k}}\left[i\right],\forall m\in \left\{3,4\right\}$ and $y_{2\bar{k},l}\left[i\right],\forall l\in \left\{L,N\right\}$ to further simplify the expression. Then, the expected rate in the right-hand side of (50) can be minimized as

(67) $$\frac{1}{BA[u][i]}E\;\left[R_{U_{\bar{k}}}\left[i\right]\right]=\frac{1}{x_{3\bar{k}}\left[i\right]}\log_{2}\;\left(1+\gamma_{2,U_{\bar{k}}}\;y_{2\bar{k},L}^{-1}\left[i\right]\right)+\frac{1}{x_{4\bar{k}}\left[i\right]}\log_{2}\;\left(1+\gamma_{2,U_{\bar{k}}}\mu \;y_{2\bar{k},N}^{-1}\left[i\right]\right)$$

with additional constraints

(68) $$\left\{\begin{array}{c} x_{3\bar{k}}\left[i\right]\geq 1+a\;e^{-b[\theta_{2,\bar{k}}\left[i\right]-a]},\\ x_{4\bar{k}}\left[i\right]\geq 1+\frac{1}{a}e^{b[\theta_{2,\bar{k}}\left[i\right]-a]},\\ y_{2\bar{k},l}\left[i\right]\geq {\left(∥q\left[i\right]-w_{\bar{k}}{∥}^{2}+h^{2}\right)}^{\alpha_{l}/2},\;\;l\in \left\{L,N\right\}. \end{array}\right.$$

Due to the highly complex variables in (68), it is difficult to directly solve them. Therefore, in order to further handle the complex variables in (68), we introduce new relaxation variables $z_{3\bar{k}}\left[i\right]$, $z_{4\bar{k}}\left[i\right]$ and obtain the following new constraints.

Since given any A>0, the function $f\left(x,y\right)=\frac{1}{x}\log_{2}\left(1+\frac{A}{y}\right)$ is jointly convex with respect to its positive variables, we can prove that all terms in $\frac{1}{BA[u][i]}E\left[R_{U_{k}}\left[i\right]\right]$ are convex with respect to their corresponding variables. Thus, by applying the first-order Taylor expansion at any given point, $\frac{1}{BA[u][i]}$$E[R_{U_{k}}\left[i\right]]$ can be approximated by its global lower bound as below:

(69) $$\begin{array}{cc} \; & \frac{1}{BA[u][i]}E\left[R_{U_{k}}\left[i\right]\right]\geq \frac{1}{x_{3k}^{r}\left[i\right]}\log_{2}\left(1+\frac{\gamma_{2,U_{k}}}{y_{2k,L}^{r}\left[i\right]}\right)\\ \; & \;+\frac{1}{x_{4k}^{r}\left[i\right]}\log_{2}\left(1+\frac{\gamma_{2,U_{k}}\mu }{y_{2k,N}^{r}\left[i\right]}\right)\\ \; & \;-\frac{1}{{\left(x_{3k}^{r}\left[i\right]\right)}^{2}}\log_{2}\left(1+\frac{\gamma_{2,U_{k}}}{y_{2k,L}^{r}\left[i\right]}\right)\left(x_{3k}\left[i\right]-x_{3k}^{r}\left[i\right]\right)\\ \; & \;-\frac{1}{x_{3k}^{r}\left[i\right]}\frac{\gamma_{2,U_{k}}}{y_{2k,L}^{r}\left[i\right]ln2\left(y_{2k,L}^{r}\left[i\right]+\gamma_{2,D_{k}}\right)}\left(y_{2k,L}\left[i\right]-y_{2k,L}^{r}\left[i\right]\right)\\ \; & \;-\frac{1}{{\left(x_{4k}^{r}\left[i\right]\right)}^{2}}\log_{2}\left(1+\frac{\gamma_{2,U_{k}}\mu }{y_{2k,N}^{r}\left[i\right]}\right)\left(x_{4k}\left[i\right]-x_{4k}^{r}\left[i\right]\right)\\ \; & \;-\frac{1}{x_{4k}^{r}\left[i\right]}\frac{\gamma_{2,D_{k}}\mu }{y_{2k,N}^{r}\left[i\right]ln2\left(y_{2k,N}^{r}\left[i\right]+\gamma_{2,U_{k}}\mu \right)}\left(y_{2k,N}\left[i\right]-y_{2k,N}^{r}\left[i\right]\right)\\ \; & =E^{lb}\left[R_{U_{k}}\left[i\right]\right] \end{array}$$

Collecting (48)–(50), (52)–(58), (61)–(62), (64)–(66), and (67)–(68), we obtain the following convex program:

(P1.6) Convexified trajectory subproblem:

(70) $$\max_{Q,\Lambda ,\Omega ,X,Y,Z,V}\;\eta$$

subject to

(71) $$\frac{1}{I}\sum_{i=2}^{I}w_{k}\left[i\right]\geq \eta ,\;\forall k.$$

(72) $$w_{k}\left[i\right]\leq A\left[d\right]\left[i\right]B\left(\Omega_{5,k}\left[i\right]+\Omega_{6,k}\left[i\right]\right),\;\forall i\in I.$$

(73) $$\sum_{n=2}^{i}A\left[d\right]\left[n\right]B\left(\Omega_{5,k}\left[n\right]+\Omega_{6,k}\left[n\right]\right)\leq \sum_{n=1}^{i-1}A\left[u\right]\left[n\right]B\;E^{lb}\;\left[R_{U_{\bar{k}}}\left[n\right]\right].$$

and (1), (2), (3), and (4) also apply. Here, $\Lambda ={\left\{\Lambda_{5}\left[i\right],\Lambda_{6}\left[i\right]\right\}}_{i=1}^{I}$, $\Omega ={\left\{\Omega_{5,k}\left[i\right],\Omega_{6,k}\left[i\right]\right\}}_{i=1}^{I}$, $X={\left\{x_{mk}\right\}}_{m=1}^{4}$, $Y={\left\{y_{jkl}\right\}}_{j\in \{1,2\},\;l\in \{L,N\}}$, $Z=\{z_{1k},z_{2k},z_{3k},z_{4k}\}$, and $V={\left\{v_{5}\left[i\right],v_{6}\left[i\right]\right\}}_{i=1}^{I}$. The convex problem (P1.6) can be solved efficiently by CVX.

### 5.4. Overall Iterative Algorithm

Based on the above explanation, we obtain the final iterative Algorithm 1 as follows.
**Algorithm 1:** Overall iterative algorithm for problem P1**Input**: $P^{r},Q^{r},r=0$ **Result**: Solving optimization problem P1 
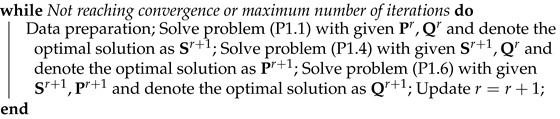



#### 5.4.1. Computational Complexity

Let *K* denote the number of ground nodes and *I* the number of time slots. Denote by $N_{S}$, $N_{P}$, and $N_{Q}$ the decision-variable dimensions of subproblems (23), (36), and (70), respectively. From the formulations in (23)–(25), (36)–(39), and (70)–(73), we can characterize the scaling of these dimensions as follows.

For the scheduling block, problem (P1.1) in (23) optimizes the scheduling variable S (e.g., the downlink/uplink indicators A[d][i] and A[u][i]) over *I* slots subject to the average-rate and information-causality constraints (24) and (25). Hence, the number of scalar decision variables in this block grows linearly with *I*, i.e., $N_{S}=\Theta \left(I\right)$.

For the power block, problems (P1.2)–(P1.4) introduce the uplink/downlink powers $\left\{p_{D_{k}}\left[i\right],p_{U_{k}}\left[i\right]\right\}$ and the slack variables $\left\{w_{k}\left[i\right]\right\}$ in (34)–(35) for all users and slots. The SCA-based convexification in (42) and (65) does not increase the order of the variable count, since it only replaces the non-convex expressions in (38) by their affine surrogates while keeping the same variable set (P,w) in (44)–(47). Consequently, the power block dimension satisfies $N_{P}=\Theta \left(KI\right)$.

For the trajectory block, problem (P1.5) in (48) optimizes the UAV horizontal trajectory $Q={\left\{q\left[i\right]\right\}}_{i=1}^{I}$, whose dimension is proportional to the number of slots *I*. In order to convexify the non-convex LoS/NLoS dependence on the elevation angles and distances, the auxiliary variables X, Y, Z, Λ, Ω, and V are introduced in (52)–(58), (61)–(62), (64)–(66), and (67)–(68). Each of these sets has cardinality proportional to KI, and the lower-bound approximation $E^{lb}\left[R_{U_{k}}\left[i\right]\right]$ in (69) only involves first-order expansions without additional optimization variables. Therefore, the overall trajectory subproblem dimension also scales as $N_{Q}=\Theta \left(KI\right)$. Summarizing, we have

(74) $$N_{S}=\Theta \left(I\right),\;N_{P}=\Theta \left(KI\right),\;N_{Q}=\Theta \left(KI\right).$$

Using interior-point methods for the convex programs (23), (36), and (70) (as employed by CVX), the worst-case arithmetic complexity of solving each block scales cubically with its variable dimension. Denoting the per-block complexities by $C_{S}$, $C_{P}$, and $C_{Q}$ for the scheduling, power, and trajectory subproblems, respectively, we obtain

(75) $$C_{S}=O\left(N_{S}^{3}\right)=O\left(I^{3}\right),\;C_{P}=O\left(N_{P}^{3}\right)=O\;\left({\left(KI\right)}^{3}\right),\;C_{Q}=O\left(N_{Q}^{3}\right)=O\;\left({\left(KI\right)}^{3}\right).$$

Let $R_{\max}$ denote the maximum number of outer BCD iterations, where in each iteration we sequentially solve the scheduling subproblem (23) and the SCA-based convex power and trajectory subproblems (44)–(47) and (70)–(73). The inner SCA loops for the power and trajectory blocks entail solving convex programs of the same order as (36) and (70), and their iteration counts can be absorbed into $R_{\max}$. Hence, the overall worst-case complexity of the proposed algorithm is

(76) $$C_{\mathrm{overall}}=O\;\left(R_{\max}\;\left(C_{S}+C_{P}+C_{Q}\right)\right)=O\;\left(R_{\max}\;{\left(KI\right)}^{3}\right),$$

where the dominant cost arises from the power and trajectory subproblems whose dimensions grow linearly with both the number of users *K* and the number of slots *I*.

Finally, the memory requirement is dominated by the storage of the KKT system factorizations in the interior-point solver, which scales as

(77) $$O\;\left(N_{S}^{2}+N_{P}^{2}+N_{Q}^{2}\right)=O\;\left({\left(KI\right)}^{2}\right),$$

and is therefore quadratic in the aggregate problem dimension.

#### 5.4.2. Convergence and Tightness Analysis

It is important to address the physical interpretation of the introduced auxiliary variables (e.g., $\left\{x_{mk}\right\}$ for elevation angle terms and $\left\{y_{jkl}\right\}$ for path loss terms) at the converged solution. Although we relax the equality definitions of these variables to inequality constraints (e.g., $y_{2\bar{k},l}\left[i\right]\geq (∥q\left[i\right]-w_{\bar{k}}{{∥}^{2}+h^{2})}^{\alpha_{l}/2}$ in (68)) to facilitate convex optimization, the inequalities hold with equality at the optimal solution. This is fundamentally guaranteed by the monotonicity of the objective function. Specifically, inspecting the reformulated rate expressions in (52) and (67), the objective function η is monotonically decreasing with respect to the auxiliary variables $x_{mk}$ and $y_{jkl}$. Consequently, to maximize the average rate η, the optimization algorithm naturally drives these variables to their lower bounds. Thus, at convergence, the auxiliary variables “tighten” to their active boundaries, ensuring that they consistently represent their meaningful physical quantities (i.e., the exact path loss and LoS probability factors) rather than acting as loose mathematical upper bounds.

## 6. Numerical Results

In this section, we present numerical simulation results to analyze the performance of the proposed UAV-assisted two-way relay system based on the PrLoS channel model. To ensure reproducibility of the numerical evaluations, all simulations were conducted on a local workstation equipped with a 13th Gen Intel^®^ Core™ i7-13700KF processor and an NVIDIA GeForce RTX 4070 GPU, which provides hardware acceleration for large-scale matrix operations. The system is configured with 32 GB of DDR5 memory running at 6000 MT/s, supplying sufficient computational headroom for solving optimization subproblems across multiple users and time slots. The simulations were executed under a stable desktop operating system, using MATLAB together with the required optimization toolkits to implement the complete evaluation pipeline. The specific configurations are shown in Table 3.

We consider the following benchmark scenarios and compare different parameters and settings through simulation experiments to validate the performance of our proposed algorithm by jointly optimizing variables S, P, Q under PrLoS channel (proposedscheme). The following three scenarios are included:

- SPQ-L: Joint optimization of variables S, P, Q under LoS channel.
- SQ-PL-FP: Joint optimization of variables S, Q under PrLoS channel with fixed P.
- PQ-PL-FS: Joint optimization of variables P, Q under PrLoS channel with fixed S.

Specifically, in SPQ-L, the average path loss of the channel is set to 2 and $P_{k}^{L}\left[i\right]=1,$∀k∈K,∀i∈{1,2,…,I}. In SQ-PL-FP, under the PrLoS channel model, the transmission power of the UAV and ground users is fixed at 0.05 W. In PQ-PL-FS, under the PrLoS channel model, no communication scheduling optimization is performed, and each row of A is set to $\frac{1}{l}$.

In the following simulation, we consider the two-pairs-of-users and multiple-pairs-of-users scenarios, respectively, in which the UAV performs its mission in the range 200 m × 800 m. In the two-pairs-of-users case, it is located at ${\left(200,150\right)}^{T}$, ${\left(200,30\right)}^{T}$, ${\left(600,150\right)}^{T}$, and ${\left(600,30\right)}^{T}$. The heights are all *h* = 100 m. In the multiple-pairs-of-users scenarios, we consider cases with one, two, three, four, five, and six pairs of users, respectively, and conduct simulation evaluations for each scenario using the proposed algorithm in this paper. And other simulation parameters are detailed in Table 4.

### 6.1. Two-Pairs-of-Users Case

**Figure 3:**
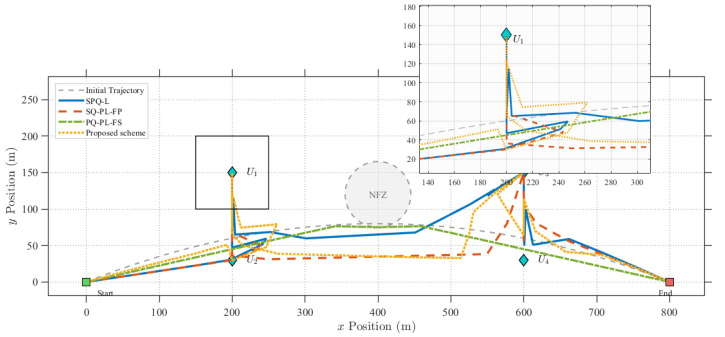
The optimized trajectories of the UAV under various benchmark schemes.

Figure 3 illustrates the optimized trajectories of the UAV under various benchmark schemes. It can be observed that, over time, the UAV in the proposed scheme initially flies towards User 2. However, rather than proceeding to User 1 via a straight path, it maneuvers towards User 1 along a curved trajectory. This behavior is rational because the trajectory, effectively optimized by the algorithm, maintains an appropriate elevation angle between the UAV and the GU_*k*_. This increases the LoS probability, enabling the UAV to maintain a maximum elevation angle with respect to GU_*k*_ for an extended duration while minimizing path loss. Consequently, this maximizes the data transmission rate within the limited mission time.

In contrast, the SPQ-L scheme dictates that the UAV fly directly above the user, hover for a specific duration, and then depart to serve the next group of users. As this approach fails to effectively account for LoS probability, the data transmission rate is reduced. Although the optimized trajectory of SQ-PL-FP resembles that of the proposed algorithm, its performance is inferior to the SPQ-PL scheme due to a lack of reasonable resource and power optimization. Furthermore, the SQ-PL-FS scheme merely steers the trajectory away from no-fly zones due to ineffective resource scheduling, resulting in no significant improvement in the data transmission rate.

**Figure 4:**
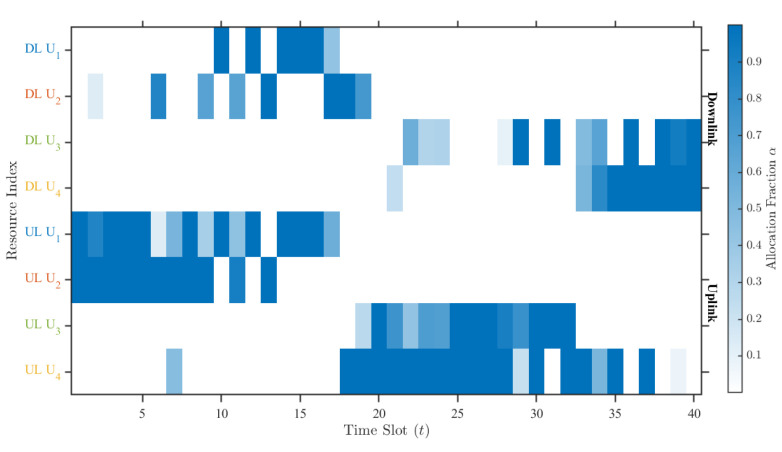
SPQ-PL RSF scheduling.

Figure 4 illustrates the RSF optimization results under the proposed scheme for a mission duration of T=40s. It can be observed that during the initial phase of the mission, both User 1 and User 2 are located relatively close to the UAV. Consequently, to maximize overall service efficiency, a significant number of time slots are allocated to User 1 and User 2 for data forwarding. As the UAV maneuvers towards User 1, a small portion of time slots is also allocated to User 3 and User 4 for data uploading. This allocation is rational, as the UAV moves closer to User 3 and User 4, demonstrating the effectiveness of the resource scheduling strategy. In the later stages of the flight, as the UAV approaches User 3 and User 4, they are allocated a larger proportion of time slots to enhance the overall data transmission efficiency.

**Figure 5:**
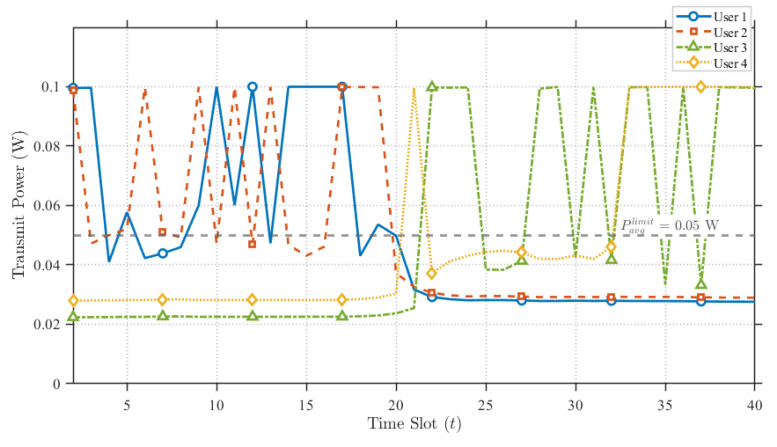
Proposed Scheme UAV downlink power allocation.

Figure 5 displays the transmission power optimization results of the proposed scheme for a mission duration of T=40s. It can be observed that during the initial phase of the mission—consistent with the time slot allocation shown in Figure 4, User 1 and User 2 are assigned a significant amount of resources. Consequently, the UAV’s downlink transmit power for User 1 and User 2 is rationally adjusted to the peak level. Although the power is occasionally reduced to lower levels, this behavior is reasonable; due to information causality constraints, once the buffered data has been effectively transmitted, lower power levels are sufficient for the remaining data transmission. Furthermore, to mitigate interference between the dual antennas, the transmit power for User 3 and User 4 is maintained at a consistently low level during this early stage. Conversely, in the later stages of the mission, the reverse pattern is observed.

**Figure 6:**
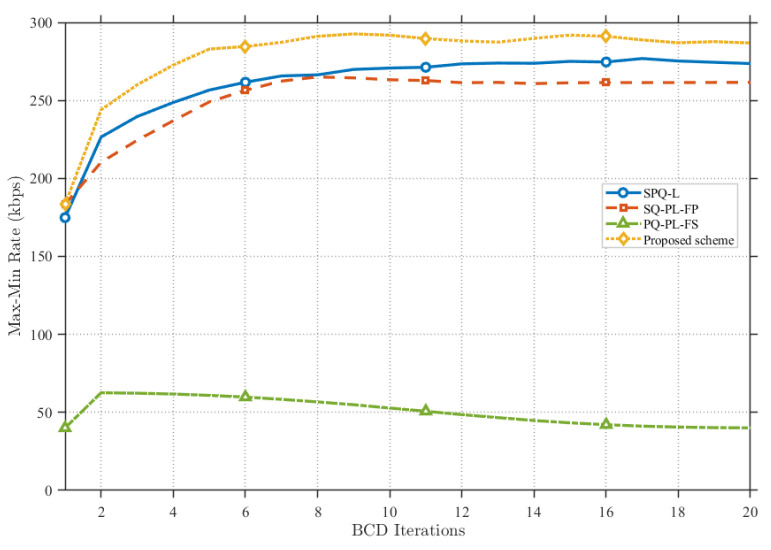
Max-min rate under different benchmark schemes.

Figure 6 compares the convergence performance of the proposed scheme against various benchmark schemes. It can be observed that the proposed scheme converges at approximately the eighth iteration. Consequently, the method demonstrates rapid convergence capabilities and robust performance. Furthermore, by effectively integrating the environmental LoS probability into the optimization problem, the proposed scheme achieves an overall system performance that significantly outperforms the benchmark schemes. In contrast, the SQ-PL-FP and SQ-PL-FS schemes exhibit relatively poor performance due to their lack of effective optimization regarding power allocation and resource scheduling.

Given that regional restrictions imposed by NFZs are introduced in this paper, we further conduct a sensitivity analysis regarding the NFZ settings during the UAV mission.

**Figure 7:**
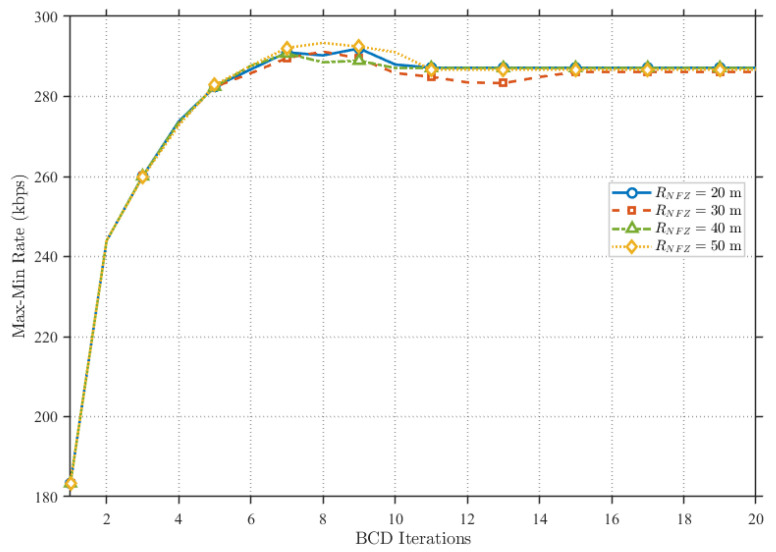
Sensitivity analysis of different NFZ sizes under the proposed scheme.

As shown in Figure 7, we adjusted the size of the NFZ within a four-user environment by setting the NFZ radius to 20m, 30m, 40m, and 50m, respectively. It is evident that when the NFZ location remains constant, varying the radius size has a negligible impact on the overall system performance. Consequently, given a fixed NFZ location, the proposed scheme is insensitive to the size of the restricted area.

**Figure 8:**
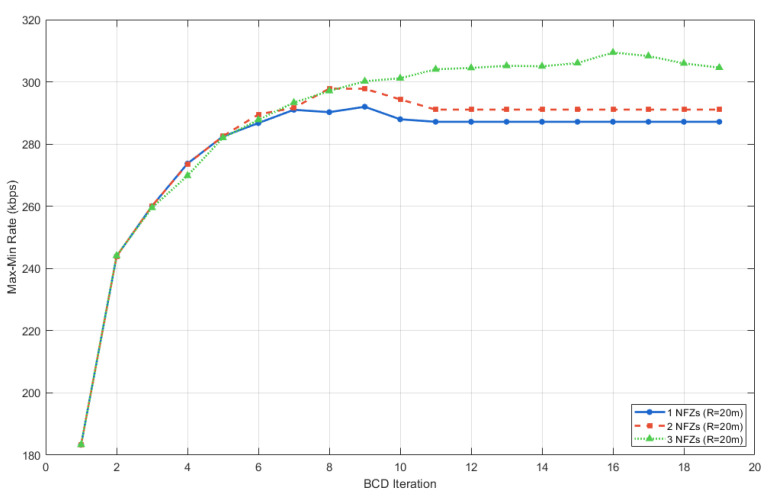
Sensitivity analysis of different NFZ numbers under the proposed scheme.

As illustrated in Figure 8, we fixed the radius of the no-fly zones (NFZs) at 20m and adjusted the number of NFZs to 1, 2, and 3, respectively. In the single-NFZ scenario, the center is located at (400,120). For the two-NFZ scenario, a second center is added at (500,140), and for the three-NFZ scenario, a third center is introduced at (550,100). It is evident that as the number of NFZs increases, the converged communication rate exhibits an upward trend, albeit with marginal variations.

Analyzing the NFZ locations in conjunction with the characteristics of the proposed method reveals that the optimization process prioritizes satisfying NFZ constraints. This prioritization can yield two distinct outcomes: it may restrict access to positions with high LoS probabilities, thereby limiting system performance. Alternatively, it may compel the UAV to maintain a trajectory characterized by high LoS probabilities—potentially overriding the dominance of standard time-slot resource scheduling which can inadvertently enhance the overall optimization objective. Consequently, we conclude that while the proposed scheme is robust to radius changes, it remains sensitive to both the location and the quantity of the NFZs when the radius is fixed.

**Figure 9:**
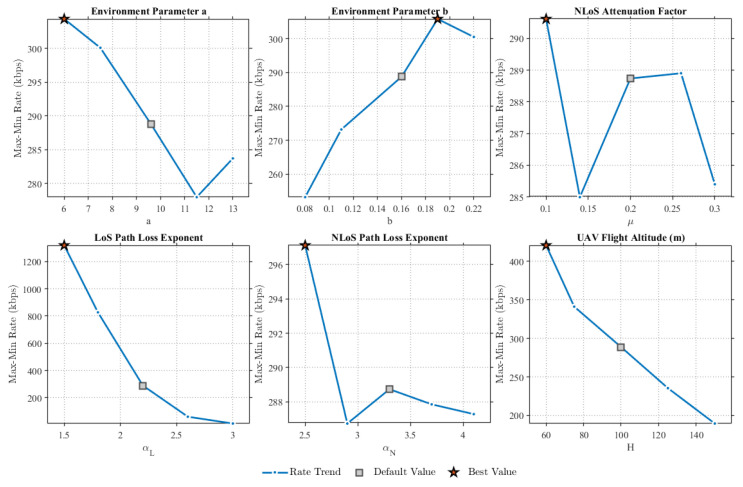
Sensitivity analysis of environmental parameters under the proposed scheme.

This paper further analyzes the impact of the involved environmental parameters. As illustrated in Figure 9, we conduct a sensitivity analysis with respect to *a*, *b*, μ, $\alpha_{L}$, $\alpha_{N}$, and the UAV flight altitude *H*. First, as *a* increases, the overall data rate exhibits a downward trend; however, since the variation is limited to the range of 20 to 30kbps, the proposed scheme demonstrates low sensitivity to parameter *a*. Conversely, as *b* increases, the overall rate shows an upward trend with a variation between 40 and 50kbps, indicating that the scheme possesses moderate sensitivity to parameter *b*. In contrast, the performance with respect to μ exhibits continuous fluctuations with a minimal overall amplitude, implying that the proposed scheme is insensitive to parameter μ. Further analysis of $\alpha_{L}$ reveals a substantial range of rate variation, whereas the variation for $\alpha_{N}$ is negligible; therefore, the proposed scheme is highly sensitive to $\alpha_{L}$ and insensitive to $\alpha_{N}$. Finally, as the UAV altitude increases, the range of rate variation is also significant, demonstrating that the proposed scheme is highly sensitive to the UAV’s altitude. Consequently, an appropriate altitude must be selected for mission execution.

**Figure 10:**
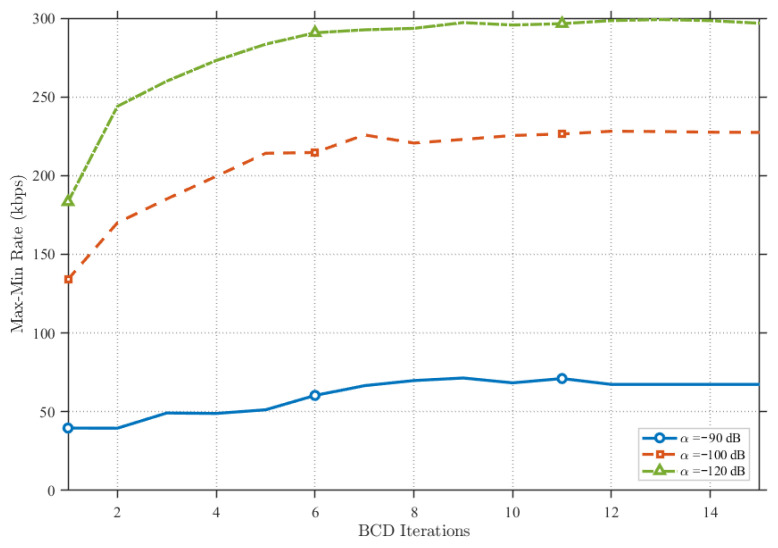
Comparison of the proposed scheme under different RSI levels.

Figure 10 presents a comparison under varying degrees of RSI. It can be observed that as the RSI suppression capability increases, the proposed scheme significantly enhances system performance. Conversely, at lower levels of RSI suppression, performance declines notably. However, as illustrated, at a moderate suppression level (specifically, α=−100dB), the overall system performance is effectively sustained through power optimization. Consequently, the proposed method is demonstrated to substantially boost performance in UAV relaying systems with robust RSI suppression while effectively mitigating the impact of self-interference in systems with limited suppression capabilities.

### 6.2. Multiple-Pairs-of-Users Case

**Figure 11:**
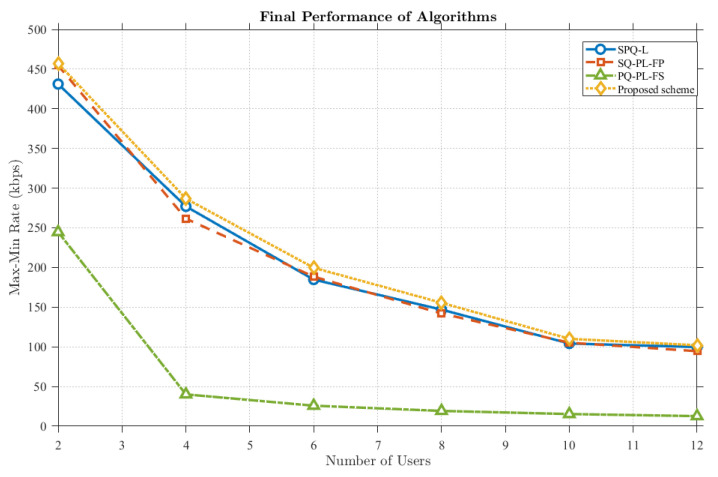
Comparison of proposed scheme rates for different users and different benchmark schemes.

As illustrated in Figure 11, it can be observed that the transmission rate exhibits a decreasing trend as the number of users increases. This decline is attributed to throughput limitations inherent in the DAFD mode, indicating that under constraints of limited antenna resources, the average gain diminishes as the number of served users grows. Furthermore, the proposed scheme consistently maintains a performance level superior to that of other benchmark schemes. This demonstrates that even in scenarios involving multiple user pairs, the proposed scheme remains capable of providing enhanced gain.

**Figure 12:**
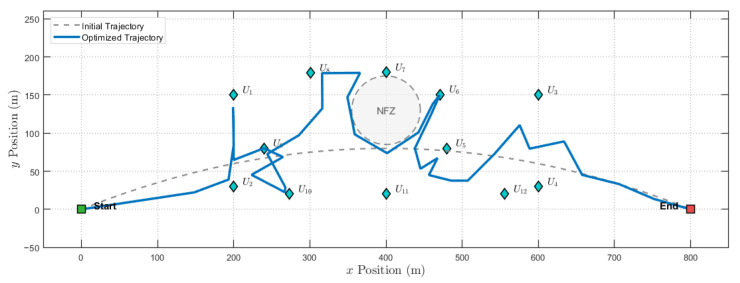
The optimized trajectories of the UAV under 12 users with proposed scheme.

Figure 12 illustrates the optimized UAV trajectory under the proposed scheme. To serve multiple pairs of users, the UAV shuttles between the users, providing effective communication services while successfully avoiding the NFZ.

## 7. Conclusions

This paper proposes a two-way UAV relay system framework based on a probabilistic LoS channel model and designs a communication protocol to maximize the minimum average rate of ground users. By optimizing RSF, transmission power, and UAV trajectory, an efficient offline algorithm combining SCA and BCD methods was utilized for iterative optimization. Extensive simulations show the advantages of this paper’s scheme, demonstrating the importance of the newly designed protocol, as well as the consideration of more accurate channels, for the performance enhancement of multi-user two-way relaying UAV communications.

It is worth noting that due to the non-convex nature of the joint optimization problem, the proposed SCA-based iterative algorithm is guaranteed to converge to a sub-optimal solution (specifically, a stationary point) rather than the global optimum. Although quantifying the exact gap to the global optimum is computationally intractable for the considered system size, the first-order Taylor expansion ensures that the surrogate function is locally tight at each iteration. This guarantees monotonic improvement of the objective function, and as shown in the simulation results, the proposed scheme significantly outperforms benchmark schemes.

## Figures and Tables

**Table 1:** Summary of UAV relay research.

Reference	Channel Model	Communication Mode	Protocol Type	Service Type
[3]	LoS	Two-Way	Time Slot Pairing	Dual Users
[4]	LoS	Two-Way	Time Allocation	Multiple Users
[5]	LoS	Two-Way	Four Slots with Multiple Jumps	Dual Users
[6]	LoS	Two-Way	Physical Layer Encoding (PNC)	Dual Users
[7]	PrLoS	Two-Way	Dual UAV Collaboration	Dual Users
[8]	LoS	One-Way	No	Dual Users
[9]	LoS	One-Way	Transmission Scheduling	Multiple Users
[10]	LoS	Two-Way	TDMA	Multiple Users
[11]	LoS	Two-Way	TDMA	Multiple Users
[12]	LoS	One-Way	No	Dual Users
[13]	LoS	One-Way	TDMA with Full-Duplex	Multiple Users

**Table 2:** Mathematical notations and descriptions.

**Sym.**	**Description**	**Sym.**	**Description**
System Parameters and Coordinates			
*T*	Total time period	*I*	Number of discretized slots
δ	Duration of each slot (T/I)	I	Set of slots {1,…,I}
*j*	Number of GN pairs	K	Set of GNs {1,…,2j}
**w** *_k_*	Horizontal position of GN *k*	q[i]	UAV position at slot *i*
*h*	Fixed flight altitude	S,E	Starting and ending positions
*N*	Index of the last time slot	C	Center of no-fly zone (NFZ)
$r_{b}$	Radius of the NFZ	$\hat{V}$	Max. UAV horizontal speed
$\hat{\Delta }$	Max. distance per slot		
Channel Model and Communication			
$g_{k}^{L}\left[i\right]$	Channel gain (LoS)	$g_{k}^{N}\left[i\right]$	Channel gain (NLoS)
$P_{k}^{L}\left[i\right]$	Probability of LoS link	$P_{k}^{N}\left[i\right]$	Probability of NLoS link
$\alpha_{L}$	Path loss exponent (LoS)	$\alpha_{N}$	Path loss exponent (NLoS)
$\beta_{0}$	Ref. channel power ($d_{0}$ = 1 m)	μ	NLoS attenuation factor
$d_{k}\left[i\right]$	Distance (UAV to GN *k*)	$\theta_{k}\left[i\right]$	Elevation angle (GN *k* to UAV)
a,b	LoS probability constants	$\sigma^{2}$	Noise power spectral density
*B*	Communication bandwidth	α	RSI suppression coefficient
Protocol, Power, and Problem Formulation			
A	RSF matrix (4j×I)	A[r][i]	Scheduling factor (row *r*, slot *i*)
$p_{U_{k}}\left[i\right]$	Uplink transmit power	$p_{D_{k}}\left[i\right]$	Downlink transmit power
$R_{U_{k}}\left[i\right]$	Uplink data rate	$R_{D_{k}}\left[i\right]$	Downlink data rate
$P_{U,\mathrm{avg}}$	Avg. power budget (GNs)	$P_{D,\mathrm{avg}}$	Avg. power budget (UAV)
$P_{U,\mathrm{peak}}$	Peak power budget (GNs)	$P_{D,\mathrm{peak}}$	Peak power budget (UAV)
u,d	RSF row indices (up/down)	$k,\bar{k}$	Pair of opposing users
P	Set of all transmit powers	Q	Set of trajectory variables
S	Set of scheduling variables	O	Set of all variables {P,Q,S}
η	Min. avg. rate (objective)	E[·]	Expectation operator
Algorithm and Auxiliary Variables			
$w_{k}\left[i\right]$	Slack var. (rate/power)	ϕ(·)	Convexifying function
${\hat{E}}^{lb,r}$	Affine lower bound (iter *r*)	*r*	Iteration counter (BCD)
$\gamma_{1,F_{m}}$	SNR term (decomp.)	$\gamma_{2,F_{m}}$	SINR term (decomp.)
$x_{mk}\left[i\right]$	Relax. var. (trajectory)	$y_{jkl}\left[i\right]$	Relax. var. (distance)
$z_{mk}\left[i\right]$	Relax. var. (angle)	$v_{5},v_{6}$	Var. for log linearization
$\Omega_{5/6,k}$	Aux. var. (DL rate)	$\Lambda_{5/6}$	Log terms (DL rate)
Λ,Ω	Aux. sets (trajectory)	X,Y	Aux. sets (trajectory)
$N_{var}$	Dim. of subproblem vars	$C_{sub}$	Complexity of subproblems
$R_{\max}$	BCD iterations		

**Table 3:** Simulation platform configuration.

Component	Specification
CPU	13th Gen Intel^®^ Core™ i7-13700KF
GPU	NVIDIA GeForce RTX 4070
RAM	32 GB DDR5 @ 6000 MT/s
Operating System	Windows 11
Software Environment	MATLAB R2022b/Mosek solver Academic authorization

**Table 4:** Simulation parameters.

Symbol	Description	Simulation Value
$\hat{V}$	Maximum flight speed of the drone	50 m/s
δ	Time duration	1 s
μ	The additional signal attenuation factor	0.2
$\alpha_{L}$	Path loss exponent	2.2
$\alpha_{N}$	Path loss exponent	3.3
$\beta_{0}$	Interference channel gain	−50 dB
a,b	Path loss parameter values	9.61, 0.16
$\sigma^{2}$	Noise power on subcarriers at ground nodes	−140 dBm/Hz
$P_{U,\;\mathrm{peak}\;}$	The peak power at the GNs	0.1 W
$P_{D,\;\mathrm{peak}\;}$	The peak power at the UAV	0.1 W
α	The RSI suppression coefficient at the UAV	[−90 dB, −100 dB, −120 dB]
ϵ	Iterative precision	0.005

## Data Availability

The dataset can be made available upon request to the authors.

## Abbreviations

The following abbreviations are used in this manuscript:

UAV	Unmanned Aerial Vehicle
Agri-IoT	Agricultural Internet of Things
LAIMs	General Artificial Intelligence Large Models
BSs	Base Stations
LoS	Line of Sight
PrLoS	Probabilistic Line of Sight
NLoS	non-LoS
PNC	Physical Layer Encoding
TDMA	Time-Division Multiple Access
DDATSAP	Dynamic Dual-Antenna Time-Slot Allocation Protocol
RSF	Resource Scheduling Factor
SCA	Successive Convex Approximation
BCD	Block Coordinate Descent
GNs	Ground Nodes
GN*k*	Ground Node*k*
DF	Decode-and-Forward
OFDM	Orthogonal Frequency Division Multiplexing
PHY	Physical
AMC	Adaptive Modulation and Coding
MAC	Medium Access Control
DAFD	Dual-Antenna Full-Duplex
SPQ-PL	Joint Optimization of Variables S, P, Q under LoS Channel
SPQ-L	Joint Optimization of Variables S, P, Q under LoS Channel
SQ-PL-FP	Joint Optimization of Variables S, Q under PrLoS Channel with Fixed P
PQ-PL-FS	Joint Optimization of Variables P, Q under PrLoS Channel with Fixed S
